# Preclinical Models and Resources to Facilitate Basic Science Research on Malignant Mesothelioma – A Review

**DOI:** 10.3389/fonc.2021.748444

**Published:** 2021-11-11

**Authors:** Ben William Johnson, Ken Takahashi, Yuen Yee Cheng

**Affiliations:** Asbestos Diseases Research Institute, Sydney, NSW, Australia

**Keywords:** mesothelioma, research resources, preclinical models, facility, biobank

## Abstract

Malignant mesothelioma is an aggressive cancer with poor prognosis, predominantly caused by human occupational exposure to asbestos. The global incidence of mesothelioma is predicted to increase as a consequence of continued exposure to asbestos from a variety of sources, including construction material produced in the past in developed countries, as well as those currently being produced in developing countries. Mesothelioma typically develops after a long latency period and consequently it is often diagnosed in the clinic at an advanced stage, at which point standard care of treatment, such as chemo- and radio-therapy, are largely ineffective. Much of our current understanding of mesothelioma biology, particularly in relation to disease pathogenesis, diagnosis and treatment, can be attributed to decades of preclinical basic science research. Given the postulated rising incidence in mesothelioma cases and the limitations of current diagnostic and treatment options, continued preclinical research into mesothelioma is urgently needed. The ever-evolving landscape of preclinical models and laboratory technology available to researchers have made it possible to study human disease with greater precision and at an accelerated rate. In this review article we provide an overview of the various resources that can be exploited to facilitate an enhanced understanding of mesothelioma biology and their applications to research aimed to improve the diagnosis and treatment of mesothelioma. These resources include cell lines, animal models, mesothelioma-specific biobanks and modern laboratory techniques/technologies. Given that different preclinical models and laboratory technologies have varying limitations and applications, they must be selected carefully with respect to the intended objectives of the experiments. This review therefore aims to provide a comprehensive overview of the various preclinical models and technologies with respect to their advantages and limitations. Finally, we will detail about a highly valuable preclinical laboratory resource to curate high quality mesothelioma biospecimens for research; the biobank. Collectively, these resources are essential to the continued advancement of precision medicine to curtail the increasing health burden caused by malignant mesothelioma.

## Introduction

Malignant mesothelioma (MM) is an incurable and highly aggressive form of cancer associated with occupational or environmental exposure to asbestos; a long-established human carcinogen (1). The global incidence of MM cases, approximated by the number of deaths, has increased significantly. The most recent Global Burden of Disease (GBD) study estimated 29,000 mesothelioma deaths (2), while other researchers estimated 38,000 mesothelioma deaths each year as a consequence of the augmented and widespread use of asbestos over the last century (3). The cancer develops most commonly within the mesothelial tissue of the pleura, accounting for approximately 80% of all MM cases, and in rarer cases; the peritoneum, pericardium, and the tunica vaginalis (4). Most cases of MM develop after a long latency period; on average 40 years (ranging between 30 to 60 years following asbestos exposure, with patients being diagnosed at a mean age of 74 years (5). With few available biomarkers and treatment options, the median survival of MM patients after diagnosis is 12-18 months following first-line standard chemotherapy with cisplatin plus pemetrexed (6, 7). To address this issue, substantial research efforts have been conducted over the past years, having provided valuable insights into the carcinogenic properties of asbestos fibres and their associated molecular alterations; as well as significant preclinical studies that have provided the foundation for the development of innovative diagnostic and treatment strategies. Despite these research efforts, the diagnosis and treatment of MM remains ineffective. It is not always practical/feasible for researchers to investigate MM biology and novel diagnostic/therapeutic strategies in MM patients directly; primarily because: 1) MM is a rare cancer, meaning that few patients can be enlisted for randomised clinical trials, and 2) invasive surgical procedures are required for sampling tumour tissue, which is often not possible to perform in MM patients with deteriorating health (8). Hence, further basic science research and development of improved MM-specific biological models are needed to address the ongoing asbestos burden and current clinical limitations associated with the diagnosis and treatment of MM.

The objective of this review article is to summarise and evaluate the effectiveness of current preclinical biological models and technologies that are currently available to researchers investigating MM. Furthermore, this review will provide an overview of some of the most valuable and extensive MM-specific biobanks that are available to researchers worldwide.

## Resources for Research

High quality research into asbestos-related disease requires a well-established laboratory that is equipped with an extensive range of resources and highly trained researchers. Typical resources that are essential to an asbestos-related disease research laboratory include a repository of high quality biospecimens, known as a biobank; as well as modern laboratory facilities (e.g. biological safety cabinets for *in vitro* cell culture experiments and animal housing for *in vivo* rodent experiments), technology (e.g. next generation sequencing platforms) and established laboratory techniques (e.g. three-dimensional cell culture). These factors combined are what provide an effective foundation to support basic science research that has strong potential for translation into clinical trials and ultimately into clinical practice in order to provide improved standards of diagnosis and treatment to individuals affected by MM.

The highly aggressive asbestos-related cancer, MM, is associated with poor prognosis and is notoriously resistant to conventional cancer-based therapies. Therefore, an understanding of the biological characteristics and associated molecular pathways that drive the development and progression of MM tumours is greatly warranted. The use of a variety of preclinical models, such as cell lines, mouse models and human-derived clinical samples, are highly advantageous to research that aims to elucidate the biological mechanisms of MM. These models are also very useful for the identification of novel prognostic and diagnostic biomarkers, and for the testing of novel therapeutic strategies. The different types of biological models, techniques and technologies available to MM researchers are discussed in detail below.

### Cell Lines

Cell lines that have been established from primary human or animal cells can be propagated repeatedly under controlled conditions outside of their natural environment. They are an invaluable resource for research into disease and have led to multiple important medical-related discoveries and developments. MM cell lines have been widely utilised as an *in vitro* preclinical model by researchers to study the pathogenesis and molecular mechanisms of MM, particularly to facilitate an assessment of cellular response to novel anti-cancer agents (e.g. platinum-based chemotherapy drugs), cytokine production, response of immune effector cells, and to define various genetic and phenotypic characteristics (9).

The first human MM cell lines were established in 1982 from the abdominal fluid of a patient (10) and the first malignant pleural-derived MM cell line, H-Meso-1, was established by Reale et al. in 1987 (11). Since that study, a variety of MM cell lines have been established and characterised with over 400 currently listed in Cellosaurus (https://web.expasy.org/cgi-bin/cellosaurus/search). Stable MM cell lines have an almost unlimited growth potential and are frequently used as a preclinical tool for research due to their easy handling, manipulation and capacity to generate high-throughput data. Constant characterisation of the cell lines *via* the analysis of typical MM markers (e.g. mesothelin, calretinin, 5T4, podoplanin, cytokeratins, and HBME_1_), karyotyping and/or short tandem repeat/single-nucleotide polymorphism analysis is important to confirm that they maintain properties consistent with the original tumour subtype (8). Whilst a range of MM cell lines are commercially available, it should be noted that primary MM cells represent a better *in vitro* model given that they more closely resemble molecular and histological characteristics to those of the original tumour (12, 13). For instance, commercial MM cell lines have been reported to exhibit significant molecular and karyotypic differences in comparison to primary MM cell lines, due to the greater number of divisions associated with the continuous culture of established commercial MM cell lines (13). It has been suggested that these molecular and karyotypic discrepancies can be attributed to the generation of highly selected clonal tumour cell populations that only partially represent those comprising the original tumour (9). Hence, it has been proposed that established MM cell lines are better suited for preliminary screening studies, followed by subsequent confirmation of the experimental findings using primary cancer cells sourced from patients (14). The applications, advantages and disadvantages of both established MM cell lines and primary MM cells are summarised in **Table 1**.

**Table 1:** Summary of the types of *in vitro* and *in vivo* preclinical models of MM, their applications to research and their main advantages and disadvantages.

Preclinical model	Model Type	Application to MM research	Advantages	Disadvantages
Primary MM cells 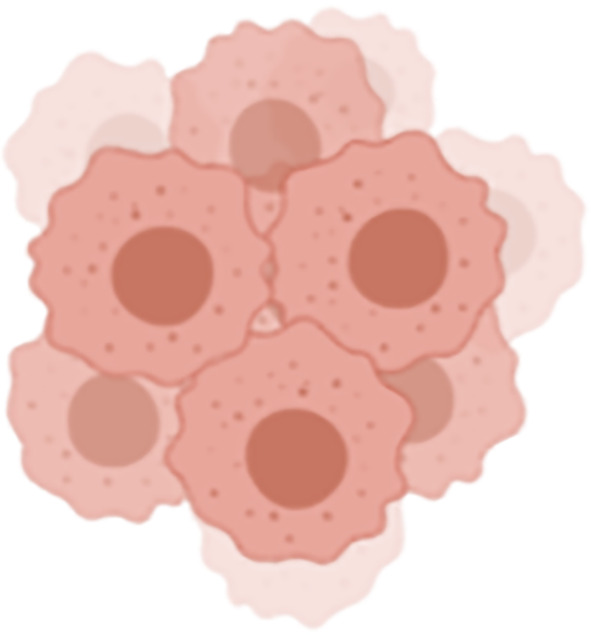	*In vitro*	*Investigating the genetic and phenotypic characteristics of MM. *Determining cellular response to novel therapeutic agents. *Identifying and/or validation of diagnostic and prognostic/predictive biomarkers.	*Cost-effective. *Easy to manipulate and handle. *Same genotypic and histological characteristics in comparison to the original MM tumour. *Absolute control of physical environment.	*Limited lifespan in culture *Very prone to contamination *Lack of 3D structure; limited cell-cell interactions; unnatural substrate
Established MM cell lines 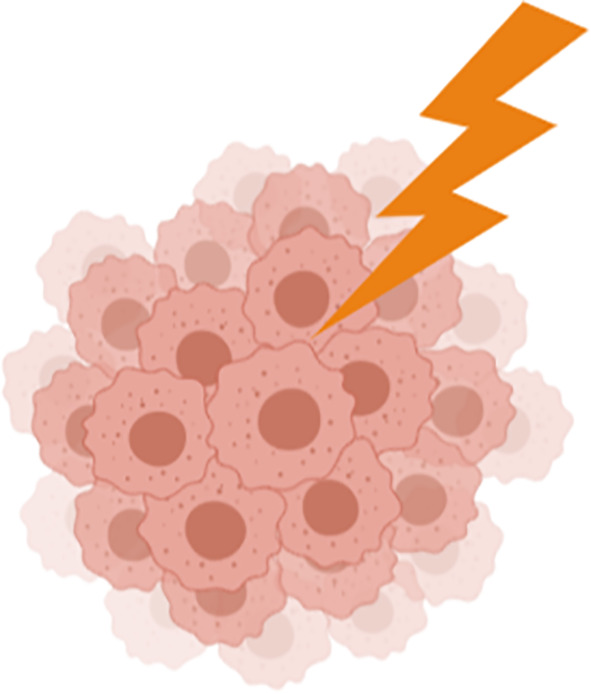	*In vitro*	*Same applications as for primary MM cells.	*Cost-effective. *Easy to manipulate and handle. *Absolute control of physical environment. *Easy to maintain. *Unlimited lifespan in culture. *High-throughput capacity.	*Cells change over time in culture (i.e. genotypic and phenotypic drifting) = reduced genotypic and histological similarities compared to the original tumour. *Lack of 3D structure; limited cell-cell interactions; unnatural substrate.
Asbestos injection 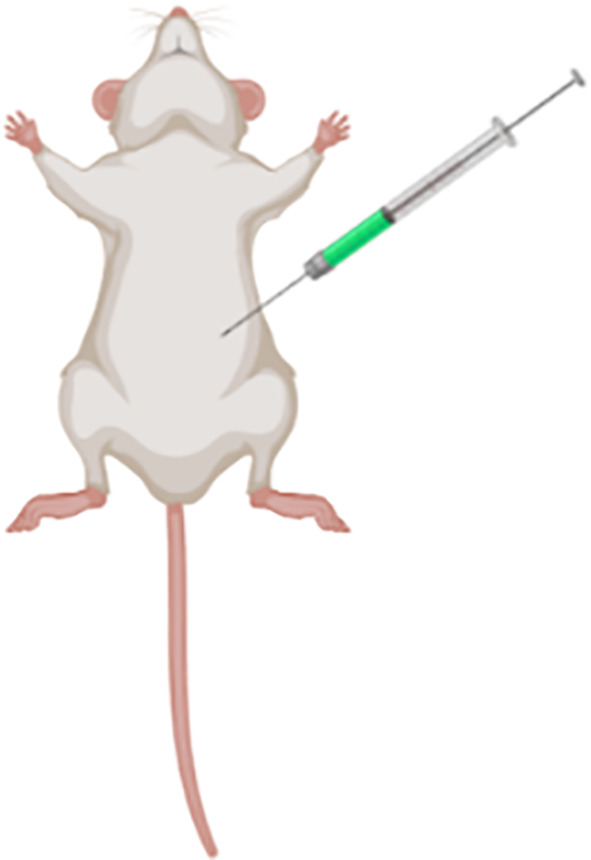	*In vivo*	*Determining pathogenic mechanisms of MM development. *Identifying early biomarkers of MM.	*Exhibits similar pathogenetic, drug sensitivity and morphological characteristics to human MM.	*Not representative of human exposure to asbestos (i.e. concentrations of asbestos fibres reaching mesothelial cells are much higher than would be expected for real-world human exposure). *Low incidence and long latency of tumour development.
Asbestos inhalation 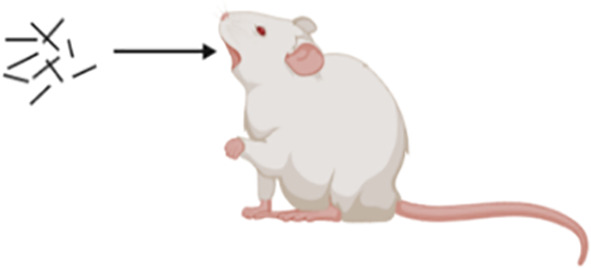	*In vivo*	*Investigating the carcinogenicity of airborne asbestos fibres. *Identifying early biomarkers of MM.	*More representative model of human exposure to asbestos.	*Requires expensive safety equipment, PPE and facilities. *Poses a greater hazard risk to staff and surrounding environment. *Not always feasible to regulate the quantity of inhaled asbestos fibres. *Molecular mechanisms/genetic traits do not always resemble that of human MM. *Low incidence and long latency of tumour development.
Cell line-derived xenografts 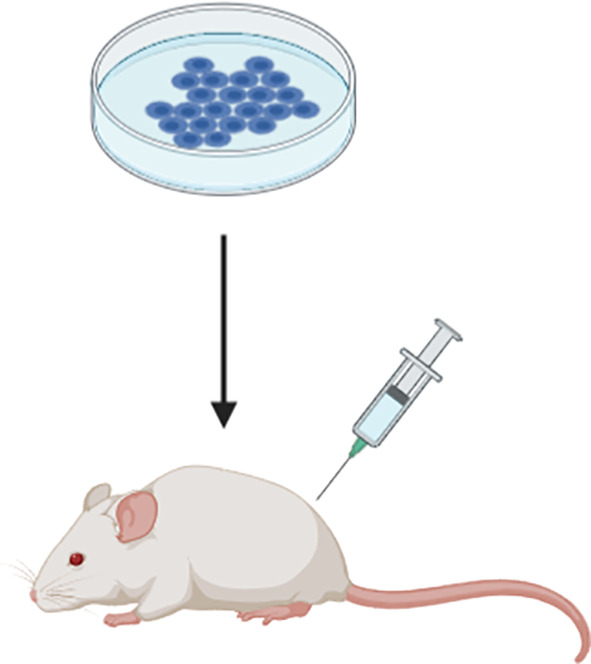	*In vivo*	*Investigating the molecular mechanisms that mediate MM tumour growth and tumour response to drug treatment. *Identification of predictive biomarkers.	*Reproducible tumour growth.	*Lack of an intact immune system means that TME does not accurately reflect that of human MM. *Not suitable for studies aiming to explore the role of immune cell populations in regards to tumour clearance and response to immunochemotherapy. *Tumours formed from cell lines do not reflect intra-tumour heterogeneity typical of human MM tumours. *TME is gradually replaced by murine cells over generations.
Patient-derived xenografts 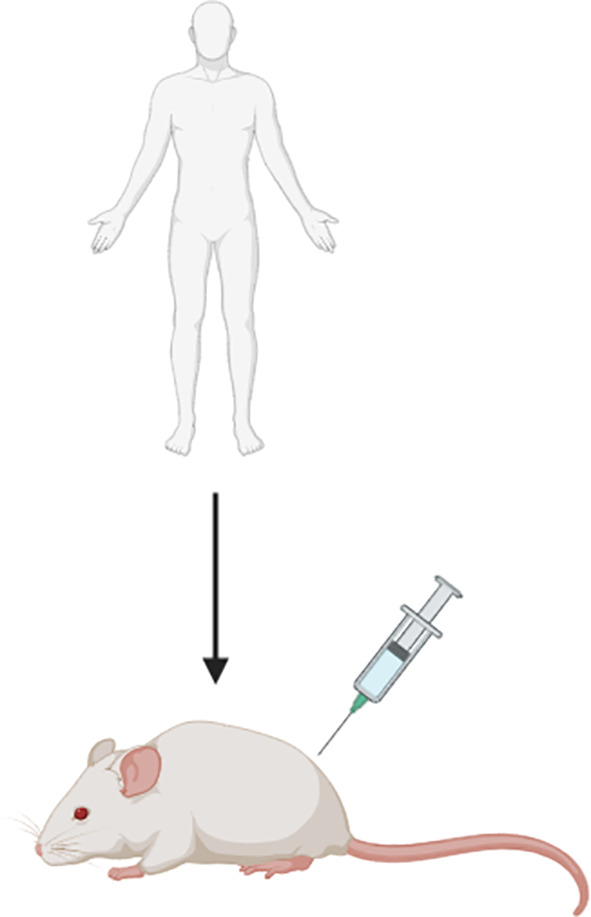	*In vivo*	*Same applications as for cell line-derived xenografts.	*Maintain the main histological features of human MM, including the stromal component. *The heterogeneity of the original tumour is at least partially preserved	*Lack of an intact immune system means that TME does not accurately reflect that of human MM. *Not suitable for studies aiming to explore the role of immune cell populations in regards to tumour clearance and response to immunochemotherapy. *TME is gradually replaced by murine cells over generations.
Syngeneic subcutaneous 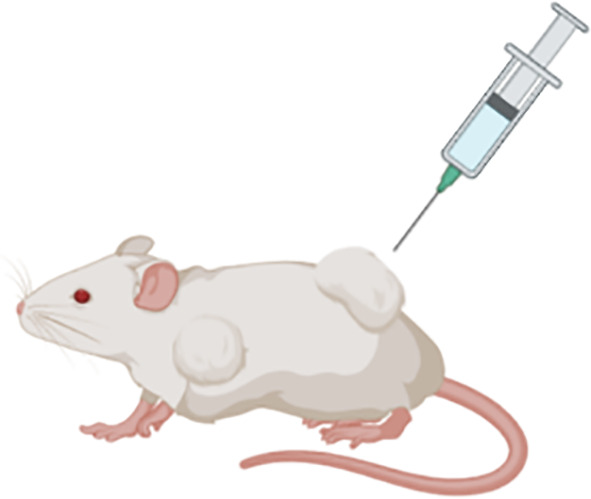	*In vivo*	*Analysing tumour growth in response to novel therapeutic agents (e.g. pharmacological studies).	*Tumour retains many histological features comparable to human MM solid tumours. *Tumour growth is generally rapid. *Tumour growth can be directly observed and measured.	*Tumour develops in an atomically irrelevant site, therefore the TME is not reflective of the human MM TME.
Orthotopic 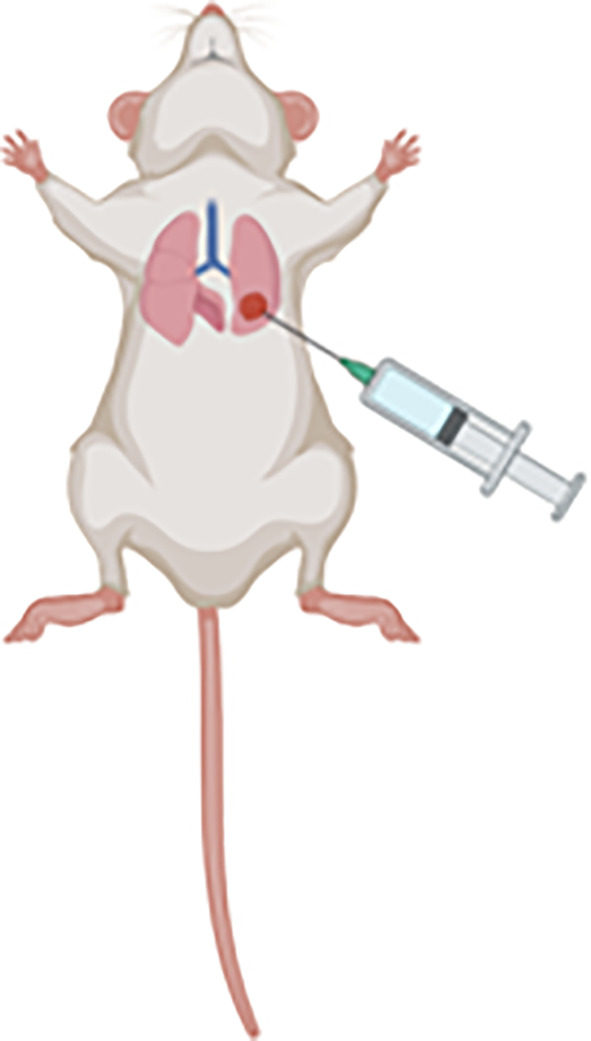	*In vivo*	*Same applications as for subcutaneous.	*Tumour develops in an anatomically relevant site. *Tumour generally grows more rapidly and invasively than the subcutaneous model. *Tumour development is influenced by the host tissue and relevant host factors such as immune system, TME, vasculature and metabolites. *Intraperitoneal models conserve similar pathological, histological, progression and response to treatment as pleural mesothelioma.	*Advanced level of technical skill/training required for intrapleural injection. *Tumour growth cannot be directly observed or measured.
Genetic predisposition 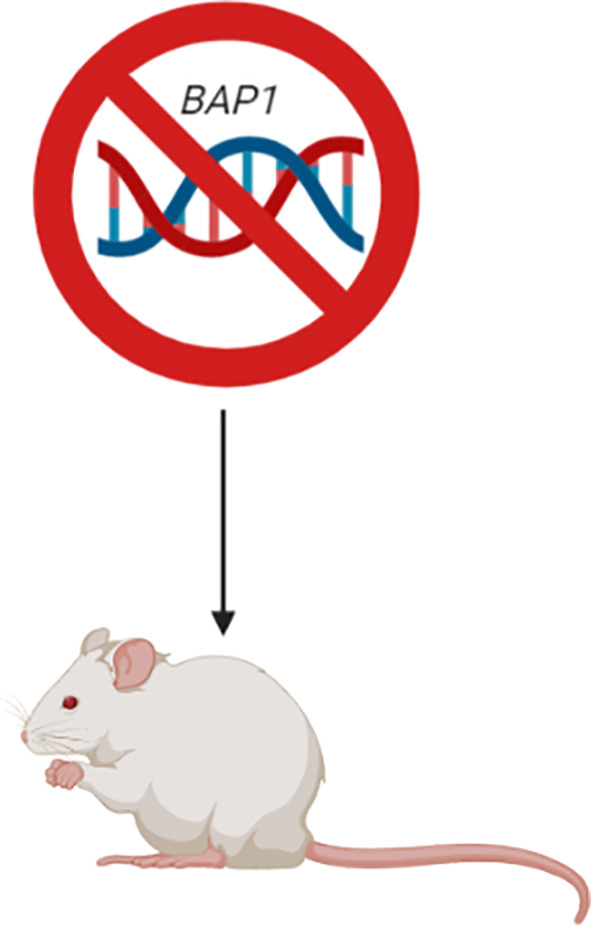	*In vivo*	*Determining the pathogenic mechanisms of MM tumour development. *Studying genetic traits that drive MM tumour development.	*Molecular characteristics of the tumour are comparable to human MM. *Higher incidence of MM development and more rapid tumour growth compared to wild type mice.	*High tendency to develop spontaneous unrelated tumours, rendering this model unsuitable for pharmacological studies. *P53 KO mice do not accurately reflect a gene mutation typically seen in human MM. *Tumour growth cannot be directly observed or measured.
MexTAg 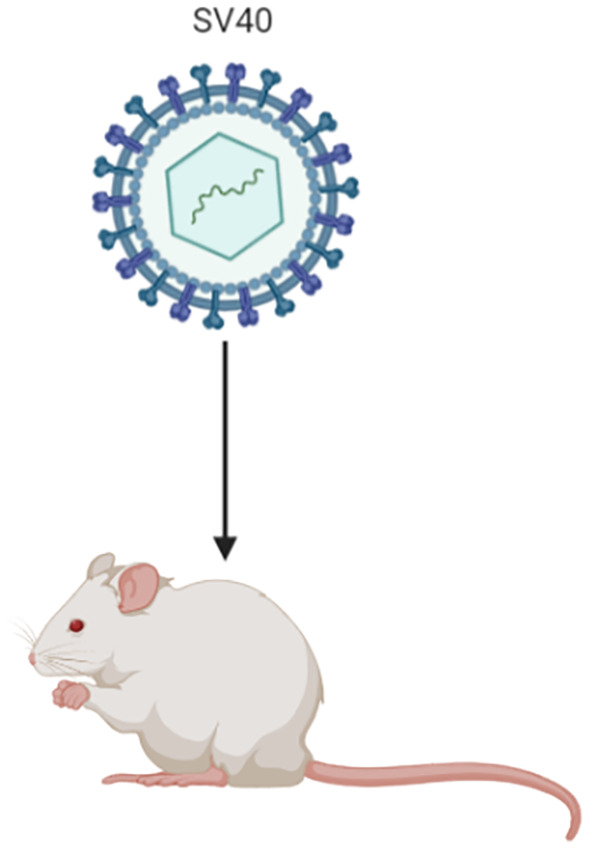	*In vivo*	*Determining the pathogenic mechanisms of MM tumour development. *Studying genetic traits that drive MM tumour development. *Analysing tumour growth in response to novel therapeutic agents (e.g. pharmacological studies).	*Guaranteed 100% incidence of MM tumour development. *Rapid, uniform and predictable disease development upon exposure to asbestos. *Exhibits similar disease pathology and response to treatment as seen in human MM. *Low incidence of unrelated tumour development.	*Tumour growth cannot be directly observed or measured. *Tumours of this model are mostly of the sarcomatoid type, which is not an accurate reflection of the more common epithelioid type seen in human MM tumours.

A number of murine MM cell lines, such as AB1, AB12, AB22, 40, 40L, AE17, and AK7, have been generated from spontaneously arising MM tumours in wild-type mice exposed to asbestos (15, 16). These cell lines display similar phenotypical and functional characteristics akin to human MM and have been widely used by researchers for *in vitro* assays or for implantation in immunocompetent mice of the same genotype for *in vivo* studies (8). Furthermore, a whole exome sequencing analysis of 15 murine MM cell lines demonstrated that murine MM has a similar mutation rate to human MM (17). This finding establishes relevance to human-based MM basic science research and justifies their continued use.

### Animal Models

Animal models are an *in vivo* preclinical model that are highly valuable in facilitating the understanding of the pathogenesis, biology and progression of MM in a living system. Additionally, animal models are useful for the development and preclinical testing of novel therapeutic drugs. The introduction of genetic mutations in rodents often results in the development of tumours that closely resemble the human disease. Hence, animal models are not only a highly valuable resource, but an important requirement for research aimed to translate novel intervention, diagnostic or treatment strategies into the clinical setting. Here we describe the applications, advantages and disadvantages of eight different types of rodent models that can be utilised for MM-based research, as also summarised in **Table 1**. These include asbestos exposure, inhalation, injection, xenograft, syngeneic subcutaneous, orthotopic, genetic predisposition and the transgenic MexTAg mouse models.

#### Asbestos Exposure, Injection and Inhalation Models

A number of studies have successfully induced MM tumour development in mice and rats *via* means of inhalation or injection of the asbestos fibres, or in hamsters through exposure to the Simian Virus 40 (SV40) (18, 19). The first asbestos exposure studies on laboratory rat models were conducted in the 1960’s, showing successful MM tumour development after intrapleural or intrathoracic (IT) injection of different forms of asbestos fibres (20). A subsequent study also conducted IT-based inoculations with amphibole and serpentine asbestos fibres in mice, however fibrosis and granulomas were more frequently observed (21). Intraperitoneal (IP) injection of asbestos fibres was therefore favoured by subsequent carcinogenicity studies in mice, which resulted in the development of MM malignancies in greater than 20% of wild type mice (22). Although peritoneal MM accounts for roughly 10% of all human MM cases, it shares similar pathogenetic mechanisms and poor drug sensitivity of the more common pleural MM (8). Furthermore, MM tumours of IP injection models were found to possess all possible morphological traits as observed in human MM (23). In contrast to injection-based MM animal models, inhalation-based models are more representative of human exposure to asbestos on the basis that they precisely emulate the human inhalation conditions, which is particularly advantageous to preclinical studies aiming to simulate the initial disease pathogenesis and/or assess the carcinogenicity of varying types of asbestos (24). The practicality of inhalation-based models is hampered by a number of factors however, including the complexity and cost of setting up exposure chambers and difficulty to control the amount of inhaled asbestos fibres. Consequently, inhalation-based animal studies require specialised safety equipment and facilities that are not widely accessible or affordable to perform in many research laboratories (8, 9). Furthermore, several studies have demonstrated a discordance in cytogenetic, gene expression and gene inactivation in inhalation-based MM rat models compared to the human MM counterpart (19, 25–27). This indicates that whilst inhalation models may closely mimic human exposure to asbestos, the biological mechanisms leading to disease pathogenesis may not necessarily reflect that of human MM. Therefore, the type of model utilised by researchers should be carefully selected depending on the objective(s) of the study. If the potential carcinogenicity of various types of airborne asbestos fibres is being investigated, then an inhalation model is probably the most appropriate model; conversely, if the various biological processes that occur post-exposure are being investigated, then an injection model would be a suitable alternative.

#### Xenograft Models

Xenograft models of MM constitute the transplantation of human solid MM tumours or cell lines into mice and are highly useful for studying molecular mechanisms that drive tumour growth and drug toxicity. Patient-derived xenograft (PDX) models are mouse models that consist of tumour biopsies or tumour cells sourced from patient pleural effusions. It has been shown that a PDX model of MM closely resemble both the histological and molecular characteristics of the primary tumour (28). All xenograft models of MM typically require the use of immunocompromised mice (i.e. mice lacking an intact immune system) so as to avoid rejection of the foreign tumour tissue or cells. This includes the hairless ‘nude’, severe combined immunodeficient (SCID) and recombination-activating gene (RAG) knockout mice; which lack T cells, both T and B cells, and adaptive immune cells, respectively (9). The main disadvantages of xenograft models is that they don’t reflect the complex tumour-immune interactions that occur in humans and therefore cannot be used for studies aiming to explore the role of the immune system in relation to tumour clearance and immunochemotherapy response (29, 30). This concept is particularly relevant to the recent open-label, randomised, phase 3 clinical study, CheckMate 743, which demonstrated a significant improvement to the overall survival of MM patients treated with the combinational immunotherapeutic treatment regimen; ipilimumab plus nivolumab. Patients subjected to this novel treatment regimen exhibited a median overall survival of up to 18 months compared to 12 months for the conventional cisplatin-pemetrexed chemotherapy treatment regimen (7, 31), and as a result ipilimumab-nivolumab was approved by the Food and Drug Administration (FDA) as a first-line combination treatment regimen for patients with unresectable MM. Ipilimumab and nivolumab are both antibodies that elicit an immune-mediated anti-tumour response upon binding to components of the immune system; specifically the cytotoxic T-lymphocyte-associated protein 4 (CTLA-4) and programmed cell death protein 1 (PD-1) receptor, respectively (32). Xenograft models are deficient in these T cell proteins. Hence, the use of an immunocompromised xenograft model of MM, such as the SCID and RAG knockout mice, would be unsuitable for use in prospective preclinical studies aiming to explore and develop this treatment regimen further. Furthermore, the transplantation of tumour cell lines to induce tumour formation in these models does not accurately reflect the intra-tumour heterogeneity of human MM tumours (14). Even in instances where human tumour tissue is transplanted, the tumour microenvironment (TME) is gradually replaced by murine cells over generations. It has therefore been suggested that the use of a humanised mouse model is a more suitable alternative for studies focused on anti-tumour immune response, whereby the mouse immune system is substituted with a human one (14). NOD SCID gamma (NSG) mice, which lack the interleukin 2 receptor gamma subunit (IL-2RG) that is involved in differentiation and function of numerous haematopoietic stem cells, are commonly utilised for this type of research (33). Whilst this model is useful for assessing anti-tumour immune response in MM, there is an associated risk of incomplete differentiation of the haematopoietic stem cells, high cost and the longer time required to attain NSG mice harbouring a human immune system that should carefully be considered by researchers wishing to utilise this model.

#### Syngeneic Subcutaneous Models

Syngeneic subcutaneous murine tumour models involve the injection of inbred mouse-derived MM tumour cells directly under the skin surface of immunocompetent mice of the same in-bred strain, which then develop into subcutaneous solid tumours. The key advantages of these models is that the MM tumours develop in the presence of an intact immune system, established tumours retain many histological features akin to human solid tumours, tumour growth is rapid, and tumour growth in response to novel therapeutics can easily be visualised and measured during the course of the experiment (34, 35). Furthermore, the tumour growth rate is highly reproducible when a controlled number of cells are inoculated (36). The main disadvantage of using this model however, is that the tumour develops in an anatomically irrelevant site and that the rapid tumour growth may impede normal stromal development and immune cell invasion (9, 35). Despite this limitation, there are chemo- and immuno-based therapies that have been successfully translated into the clinical setting using this type of model (37). It has therefore been suggested that the syngeneic subcutaneous model remains a useful tool for the purpose of studying therapeutic interventions for MM, such as immunotherapy-based assessment, as long as results are replicated using other anatomically relevant tumour-bearing models (35).

#### Orthotopic Models

Orthotopic models represent a more human-like disease model; the tumour develops in an anatomically relevant site and are usually more rapid growing and invasive than the subcutaneous model. This type of model closely resembles human MM, given that the tumour cells grow along the serosal surfaces, form nodules in the peritoneum, develop metastases, and form ascitic fluid in some cases (38, 39). Most importantly, the tumour develops with respect to the host tissue and its growth and development is influenced by relevant host factors such as the immune system, vasculature, metabolites and TME (9). Advanced technical skill is required for intrapleural orthotopic models as there is an associated risk of inducing a hemothorax and/or pneumothorax during the intrapleural injection procedure (35). Intraperitoneal models of MM are relatively easier to perform by less skilled researchers and conserve similar pathological, histological, progression and response to treatment as pleural mesothelioma (40, 41); therefore the intraperitoneal model is more commonly preferred over the intrapleural model. The main limitation associated with orthotopic models is that tumour growth cannot be directly observed or measured, however, this can be overcome *via* the use of fluorescence-based small animal imaging techniques. For example, the proliferation of cancer cells expressing the luciferin gene, that converts a substrate to emit light, can be measured to provide a reliable indicator of tumour growth (9, 35).

Given that the orthotopic model and syngeneic model possess an intact immune system and that tumour response to treatment can be monitored *in situ*, these models are particularly beneficial to researchers aiming to monitor the *in situ* progressive MM tumour regression in response to novel drug treatments; particularly immunotherapeutic agents such as the aforementioned ipilimumab and nivolumab.

#### Genetic Predisposition Models

Genetic predisposition mouse models have been developed in accordance with characteristic gene losses typically seen in human MM; primarily in the *NF2*, *BAP1* and *CDKN2a/ARF* gene loci. Such models have been established by genetically modifying them so that they no longer express these genes, either individually or in combination, commonly referred to as gene ‘knockout’ models. Although mutations of the *p53* tumour suppressor gene have only been reported in a few cases of MM and is not believed to play a role in driving MM tumour development, *p53*-deficient mice have been developed and have exhibited a higher incidence and more rapid tumour progression than wild type mice; particularly following asbestos exposure in the peritoneum (42–44). An alternative model, a heterozygous *Nf2* mouse, was first reported by Altomare et al. Upon repeated exposure of the heterozygous *Nf2* mice to asbestos, they found that these mice were notably more susceptible to MM development compared to their homozygous *Nf2* counterparts, with a reported incidence of 85% and 59%, respectively (44). Furthermore, the molecular features of the tumours were found to resemble that of human MM tumours, including activation of *Akt*; homozygous deletion of tumour suppressor genes *p16 (Ink4A), p14 (ARF)/p19(Arf)*, and *p15(Ink4B)*; and loss of the *Nf2* protein, Merlin (44). Other researchers have induced heterozygous *BAP1* mutations in mice in order to investigate the incidence of MM in humans carrying *BAP1* germline mutations, even with no known history of exposure to asbestos, as was the case for four members of a European family (45). Overall, the mutant *BAP1* mice exhibited increased susceptibility to MM following peritoneal injection of asbestos, as well as some without injection, with incidence of MM being double and median survival shorter for the *BAP1* mutant mice compared to the wild type controls (46). Thus, this model effectively demonstrated *BAP1* loss to be a key genetic driver of MM development, as well as being translatable to the *BAP1-*impaired human MM cases. Whilst these genetically modified mouse models have facilitated our growing knowledge of MM pathogenesis and associated molecular biology, unfortunately the *p53*, *Nf2* and *Bap1* heterozygous knockout mice have a high tendency to frequently develop spontaneous tumours, such as lymphomas, sarcomas and adenocarcinomas. Hence, these models have been deemed unsuitable for pharmacological studies (i.e. novel drug testing) of MM (35). To overcome this problem, Robinson et al. established a transgenic mouse model; a model highly susceptible to MM tumour development, but with a low associated incidence of other tumour types; the MexTAg mouse (47).

#### Transgenic MexTAg Mouse Model

The MexTAg transgenic mouse model of MM was developed by Robinson et al. through the engineering of mesothelial cells to express the oncogenic SV40 virus large T antigen (SV40 Tag), and has been utilised to highlight co-carcinogenicity between asbestos and SV40 (48). Whilst SV40 alone does not induce MM development in this model, its oncogenic potential facilitates a guaranteed 100% incidence of disease, rapid, uniform and predictable disease development upon exposure to asbestos (9). The MexTAg mice develop MM tumours that exhibit similar disease pathology and treatment responses to human MM (47). Another notable advantage of the MexTAg mouse model is that it has a lower chance of developing unrelated tumours in comparison to wild-type mice or the heterozygous and conditional mesothelioma knockout mouse models (35). It has been proposed that the Tag transgene does not influence the overall molecular mechanism of MM development in this model. Rather it phenocopies p16 loss, which induces the characteristic accelerated disease progression in this model following asbestos exposure (49). Furthermore, it has been suggested that the MexTAg model is a functional equivalent to human MM being that it similarly exhibits a loss of tumour suppressor genes such as *CDKN2A (P16^INK4a^/p14^Arf^)*, *NF2, BAP1* and *p53* (9). The suitability of the MexTAg mice for preclinical studies was assessed by Robinson et al., upon subjecting this mouse model to treatment with gemcitabine; a cytotoxic drug proven to exhibit some efficacy in human MM (47). The results of this study showed that the MexTAg mice treated with gemcitabine had a median survival of 48 weeks compared to 33 weeks for the untreated vehicle control. Given the strong concordance of MM response to gemcitabine of the MexTAg model to that of human MM, this study effectively demonstrated the translatability of the model to the clinical setting. It should be noted however, that most MM tumours that develop in this model are of the sarcomatoid type, which is different from the more common epithelioid type seen in humans (48). As with the orthotopic model, fluorescence-based small animal imaging techniques are required in order to monitor tumour growth in the MexTAg model.

### Human Biospecimens

Well characterised human biospecimens are an invaluable resource required for the advancement of translational research aimed to improve the diagnosis and treatment of MM. Types of human MM biospecimens include pleural, pericardial and peritoneal tumour tissue biopsy samples; as well as matched whole blood, plasma, serum, pleural effusion specimens and lymphocytes. In addition to their usefulness for the generation of primary cell cultures and transplantation into mouse models, human biospecimens are highly useful for biomarker validation research aimed to identify novel biomarkers to facilitate an understanding of cancer aetiology. Such knowledge can then be applied to the design and development of improved MM-specific diagnostic techniques and targeted therapies to provide an accurate diagnosis and improved prognosis for patients with MM. The diagnosis of MM in the clinical setting is particularly challenging due to a lack of effective diagnostic biomarkers and the requirement of an invasive percutaneous needle biopsy procedure or video-assisted thoracoscopic surgery (VATS) required to attain a definitive diagnosis (50). These procedures are not always feasible to perform on MM patients with significantly declining health and are dependent on the availability of services (e.g. trained staff and medical resources) (50). To date, a number of less-invasive blood-based biomarkers have been investigated for MM, such osteopontin and fibulin-3, however a poor associated specificity and/or sensitivity have rendered them unsuitable for clinical implementation as diagnostic and/or prognostic biomarkers of MM (51, 52). Continued use of human-derived biospecimens to identify and validate novel less-invasive biomarkers that are highly sensitive and specific for MM is greatly warranted and would represent a significant advancement for the diagnosis and treatment of MM. The use of large collections of well preserved biospecimens have proven to be particularly beneficial to the success of preclinical studies aiming to identify and validate novel less-invasive biomarkers for an accurate and/or early detection of MM. For example, a study conducted by Creaney et al. utilised pleural effusion samples collected from 1,331 MM patients, whereby it was established that effusion-derived mesothelin exhibits a 95% specificity for MM; justifying the clinical utility of pleural effusion-derived mesothelin as a biomarker to facilitate a definitive diagnosis of MM (53). Human biospecimens intended for use in downstream research applications are typically stored under strictly controlled conditions in a biobank facility, usually in a -80°C freezer or liquid nitrogen tank, to ensure sample integrity is maintained for subsequent histological, proteomic, genomic or transcriptomic analyses at a later date.

### Laboratory Techniques and Technology

The inability of early laboratory techniques and technologies to adequately reproduce the complex heterogeneity and/or tumour microenvironment (TME) of MM tumours is a major contributing factor to limiting our understanding of MM tumour biology and the non-concordant results obtained from previous preclinical studies and those from clinical studies. Promisingly, laboratory technologies and techniques are constantly evolving. It is therefore of vital importance that researchers select and apply the most up-to-date and clinically-relevant techniques and technology in order to produce data that best represents the clinical behaviour of MM as possible and provide a more comprehensive understanding of MM biology. Some of the useful modern techniques and technologies currently available to researchers include three-dimensional (3D) cell culture techniques and next generation sequencing (NGS) technologies, as described in detail below and summarised in **Table 2**.

**Table 2:** Summary of the applications of *in vitro* 2D and 3D cell culture methods for MM research and their main advantages and disadvantages.

Method	Application to MM research	Advantages	Disadvantages
2D cell culture 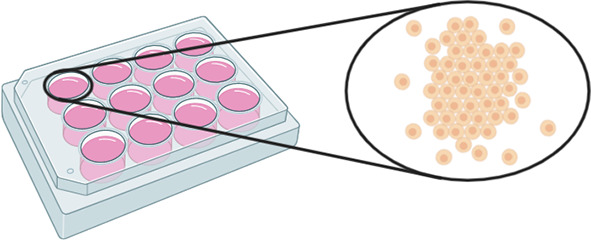	*Large scale drug testing *Identification and/or validation of novel biomarkers. *Investigating the role of genes in MM progression.	*Cost-effective *Easy handling. *Easy to maintain. *High throughput capacity.	*Drug sensitivity data generated from this method does not always reflect that of the *in vivo*/clinical counterpart. * Lack of 3D structure; limited cell-cell interactions; unnatural substrate. *Lack of cellular heterogeneity/complexity compared to the original tumour. * Gene expression less similar to *in vivo* tumours.
3D cell culture (includes spheroids, TFS and organ-on-a-chip) 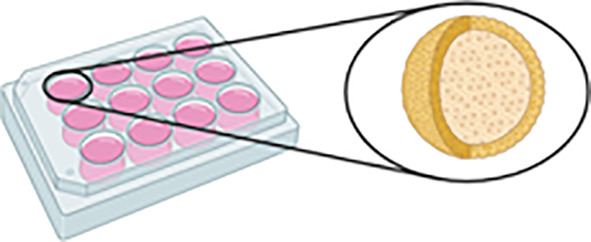	*Studying therapeutic efficacy of novel drugs. *Identification and/or validation of novel biomarkers. *Studying cell-to-cell and cell-to-extracellular matrix signaling.	*More representative of the *in vivo* tumour structure/complexity. *Gene expression more similar to *in vivo* tumours. * Drug response better reflects *in vivo*/clinical drug response. *Increased cell-to-cell and cell-to-extracellular matrix signalling.	*TFS and organ-on-a-chip require access to fresh surgical MM tumour samples = low throughput capacity. *Complex handling. *Less cost-effective.
Whole genome sequencing 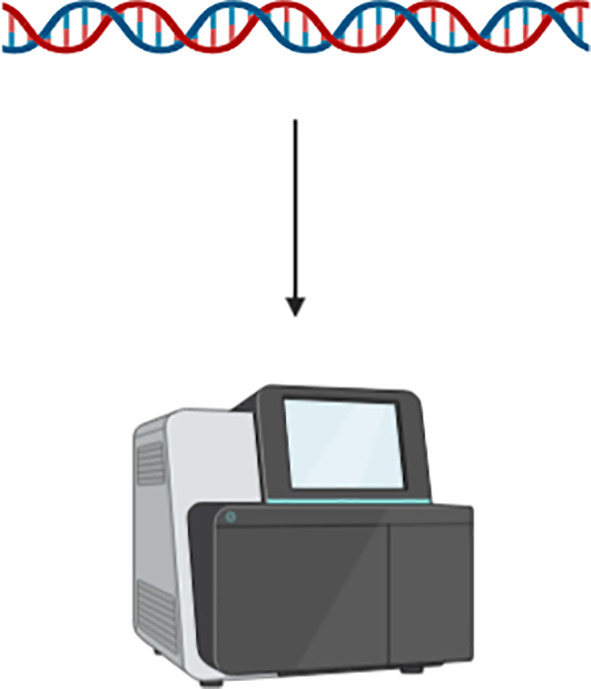	*Studying all types of MM-specific genetic variation across the entire genome.	*Detects coding, non-coding and structural variants across the entire genome.	*High associated cost. *Large volume of data to process and store. *Numerous variants of unknown significance can be detected. I.e. limited knowledge to fully understand / appreciate the significance of detected unknown variants.
Transcriptome sequencing 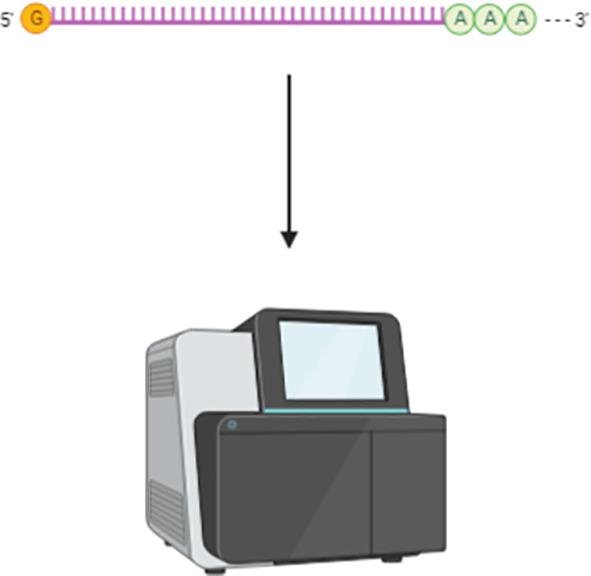	*Studying all types of aberrant MM-specific mRNA / transcript variation.	*Rapid, precise, quantitative measurement of gene expression. *High sensitivity enables detection of low-abundance transcripts. *DNA sequences can be unambiguously mapped to unique regions of the genome instead of relying on existing genome sequence data. *Useful for the discovery of single-nucleotide polymorphisms and rare mutations. *More affordable compared to whole genome sequencing.	*Transcript quantitation can be affected by biases introduced during cDNA library construction and sequence alignment. *Accurate sequence annotation and data interpretation can be computationally challenging in the absence of pre-existing reference genome(s).
Targeted sequencing 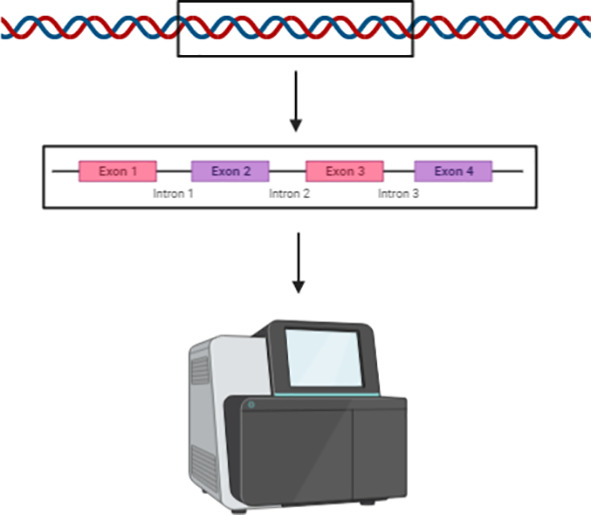	*Studying unique MM-specific alterations at the sites of specific regions of the genome (i.e. exosomes) or subset of genes.	*Significantly less time-consuming and more cost-effective than whole genome sequencing. *Specific areas of the genome can be sequenced at a greater depth than whole genome sequencing. *Reduced volume of data to process and store than whole genome sequencing.	*Only focuses on limited regions of the genome, meaning it does not take into account any other genetic variants outside of the focus/target gene panel.
Droplet digital PCR 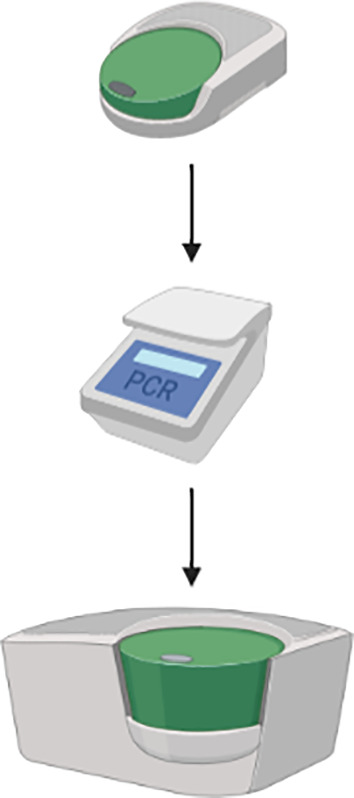	*Studying unique MM-specific gene copy number variations, DNA mutations or deletions. *Detection and validation of MM-specific biomarkers.	*Provides an absolute and independent quantification of DNA without the need for a standard curve. *Generated data is more accurate and reproducible than conventional qPCR. *Capable of detecting very low concentrated target molecules from variably contaminated samples.	*Equipment and reaction running costs are more expensive than conventional qPCR. *Requires advanced skill and handling compared to conventional qPCR.

#### 2D vs 3D Cell Culture

Cell monolayer culture, otherwise known as two-dimensional (2D) cell culture, is commonly utilised by researchers for large-scale drug testing as cells grown in this manner are easy to handle and are cost effective, however drug sensitivity data obtained from this *in vitro* model has frequently been shown to differ to their *in vivo*/clinical counterparts. MM is typically resistant to a range of chemotherapeutic drugs tested on patients in the clinical setting, however this trend is not always accurately modelled by 2D cell culture. Furthermore, drug sensitivity data derived from 2D cell culture has led to false expectations upon the subsequent testing of drugs in human clinical trials, as well as resulting in a waste of time and expenses. For instance, the proteasome inhibitor, bortezomib, was found to be highly effective in monolayer malignant pleural mesothelioma (MPM) cell line cultures (54–56), however follow-up phase II studies produced disappointing results (57, 58). To rectify this issue, recent research has led to the development and testing of 3D cell culture techniques, which more closely mimic solid tumours and their associated TME compared to 2D cell culture. There are three types of 3D models that have been developed, which includes spheroids, tumour fragment spheroids (TFS) and organ-on-a-chip.

Spheroids involve the seeding of established cell line or primary cell suspensions on 3D structures composed of an artificial matrix (i.e., polyHEMA). It has been demonstrated that spheroids acquire multicellular resistance to a variety of treatments, which more closely resembles the chemoresistance effect frequently seen in MM patients (59, 60); a trend not seen for monolayer cultures. This can most likely be attributed to the fact that some genes that mediate resistance to cell death are differentially expressed in a 3D organisation of cells compared to 2D culture (61, 62). The main limitation of this 3D model however, is the absence of other cell populations from the TME (14).

TFS constitutes an *ex vivo* model of living tumour tissue. These differ from cell-based spheroids on the basis that small fragments of the original tumour tissue are grown into 3D structures. This technique does not require an artificial matrix; rather it relies on the tumour cells’ ability to generate and self-organise complex extracellular matrix (ECM) and cell to cell interactions. TFS are highly reliable and can be utilised for many different and/or repeat experiments given that they can contain viable tumour cells for weeks to months (8). Furthermore, it has been reported that TFS retain multiple characteristics of the original tumour for up to 3 months, including the presence of viable mesothelioma cells, macrophages and a collagen-rich stroma (63).

Organ-on-a-chip is a relatively novel technology that incorporates the integration of bioengineering with microfluidics to better mimic the *in vivo* TME. Multiple tissues can be seeded within one chip, which therefore enables researchers to explore the interactions between MM cells/tissues and other host cells/tissues within a single experiment. MM tumour organoids have been developed to facilitate the screening and prediction of suitable therapeutic options that are specifically tailored to individual patients (i.e., personalised therapeutics). This was effectively demonstrated in a study by Mazzocchi et al., which showed that the MM tumour grown on a chip responded to chemotherapy that mimicked the chemotherapy-induced tumour response of the associated patient. It also demonstrated the efficacy of using the organ-on-a-chip platform to predict the effectiveness of a chemotherapy drug based on a targetable mutation specific to the tumour genotype of individual MM patients (64).

#### Next-Generation Sequencing (NGS) and Quantitative Polymerase Chain Reaction (PCR)

Various “-omics” technologies, particularly genomics and transcriptomics, have significantly improved our understanding of MM-specific gene alterations and aberrant molecular signalling. The technology enabling whole genome and transcriptome constitutes an amalgamation of discoveries and innovations in molecular biology. The introduction of the polymerase chain reaction (PCR) in 1988 enabled researchers to make numerous gene-related discoveries, until the entirety of the human genome was sequenced in 2004 (65). Since then a number of technologies, collectively called “next-generation sequencing” (NGS), have become available and increasingly accessible to researchers conducting genome-wide studies.

Massively parallel sequencing (MPS), a form of NGS, is a term used to refer to a grouping of high-throughput DNA sequencing methodologies that enable the simultaneous generation of millions of sequence reads. Such techniques are typically applied to perform whole genome sequencing, whole transcriptome sequencing, and targeted sequencing. Whole genome sequencing enables the determination of the complete human DNA sequence, and is therefore a highly useful technique for discovering a wide range of genetic variation. Transcriptome sequencing enables researchers to study the presence and quantity of RNA transcripts in a particular tissue sample at a specific timepoint, therefore, differences in gene expression and alternatively spliced gene transcripts can be identified. Targeted sequencing refers to the sequencing of a specific region of the genome (e.g. the exome) or subset of genes (66). All three of these approaches have been applied to MM, producing data that is highly useful in regards to identifying aberrant genetic variants associated with MM development and potential therapeutic targets. Examples of MPS technology/platforms that have been utilised for previous MM-based studies include the Roche/454-pyrosequencer, Illumina Genome Analyzer 2, Illumina HiSeq, Ion Torrent Personal Genome Machine, and SOLiD 5500 (66–72). The Ion Torrent platform in particular was utilised in a study by Sneddon et al. to perform whole exome and transcriptome sequencing on DNA and RNA harvested from tumour cell cultures derived from human pleural effusion samples. This study effectively determined that *BAP1, CDKN2A* and *NF2* alterations occur in pleural effusion-derived tumour cells at a higher frequency than what is typically seen in MM tumour samples, as well as identifying high frequency alterations for the *TRAF7* and *LATS2* genes. Furthermore, this study identified previously unreported alterations in the *FGFR3* gene and chromosome regions 19p13.3, 8p23.1 and 1p36.32; thus highlighting novel mutations of MM that warrant further investigation in terms of their suitability as diagnostic and/or treatment response monitoring biomarkers of MM (73). Additional novel chromosome alterations have been detected by Serio et al., whereby a high-resolution array-comparative genomic hybridisation (a-CGH) performed on peritoneal MM patient samples revealed deletions at regions 8p23.1 and 1q21; both of which were found to be co-deleted in the majority of the tested patient samples (74). Hmeljak et al. recently carried out a comprehensive analysis of 74 MM tumours as a contribution to the The Cancer Genome Atlas (TCGA), which produced valuable genomic, epigenomic, and transcriptomic data using high-throughput array and NGS technology (75). Additionally, a recent study conducted by Oey et al. utilised whole genome sequencing to effectively characterise mutations and structural alterations using DNA derived from human primary tumours and matched cultured cells (12). This study was able to establish that the majority of genetic drivers of MM are associated with structural alterations, as opposed to point mutations.

The advent of quantitative PCR (qPCR), or real-time PCR, has significantly revolutionised the way researchers quantify gene expression in biological samples. The main benefits to using qPCR over other conventional semi-quantitative PCR techniques is that they are capable of generating quantitative data at a 10,000- to 100,000-fold higher sensitivity than RNase protection assays; are able to detect a single copy of a specific transcript; can reliably detect gene expression differences as low as 23% between samples; can differentiate between different messenger RNAs (mRNAs) with nearly identical sequences; do not require post-amplification sample manipulation; and are relatively more high-throughput (76–79). The main disadvantage is that qPCR equipment and reagent running costs are considerably more expensive than standard PCR methods (79). Droplet digital PCR (ddPCR) is the most modern version of qPCR, which was made commercially available in 2011 (80, 81). As with non-digital qPCR, the ddPCR technology involves Taq polymerase in a standard PCR reaction to amplify a target DNA segment from a complex biological sample using pre-validated primer/probe assays (82). Unlike non-digital qPCR however, the ddPCR partitions the PCR reaction into thousands of individual reaction vessels prior to amplification and the data is acquired at the reaction end point. The advantage of using ddPCR over non-digital qPCR is that it provides an absolute and independent quantification of DNA without the need for a standard curve, thereby yielding more precise and reproducible data than non-digital qPCR (82, 83). Furthermore, the ddPCR can be applied to detect extremely low concentrated target molecules from variably contaminated samples, whereby the sample dilution requirements to ensure a consistent reaction efficiency, primer annealing and quantification cycle (Cq) values for non-digital qPCR would likely result in undetectable target levels (84, 85).

Most recently, we applied the ddPCR technique to a collection of serum samples obtained from MM patients, whereby the assay was optimised for the purpose of detecting circulating methylated microRNA (miR-34b/c) (86). Its degree of methylation in circulating DNA was previously reported to be associated with the development of MM (87). This study therefore effectively demonstrated that miR-34b/c is a promising biomarker candidate for predicting disease progression in patients with MM, as well as demonstrating the feasibility of ddPCR technology to detect circulating biomarkers in MM patient-derived biospecimens. We further demonstrated the utility of the ddPCR technique for MM biospecimen-derived biomarker detection using a large cohort of MM tissue samples, whereby co-deletion of the cyclin-dependent kinase inhibitor 2A (CDKN2A) and methylthioadenosine phosphorylase (MTAP) genes were detected *via* ddPCR. The homozygous loss of CDKN2A detection *via* ddPCR yielded a concordance rate of 92% with the gold standard fluorescence *in situ* hybridisation (FISH) diagnostic technique (88). Collectively these studies have highlighted that the ddPCR technique is highly reliable for MM-based research aimed to detect and validate novel biomarkers of MM, and demonstrated the potential utility of the ddPCR technique to replace or be used as an alternative to the current biopsy-reliant FISH diagnostic method.

## The Biobank

A biobank is widely defined as a facility for the collection, preservation, storage and supply of biological samples and associated data, which follows standardised operating procedures and provides material for scientific and clinical use (89). These biospecimens and data are highly valuable to scientists conducting research aimed to provide new insights into human diseases, their causes and associated molecular biology, to develop better preventative measures, and to develop improved diagnostic tests and therapies. Biobanking is usually carried out by a designated Biobank Officer; a process which is typically initiated by the Biobank Officer making contact with the patient or donor, followed by the transferal of the biospecimens and associated data to an institution that hosts the biobank. Biobanks have been established in a variety of institutions, such as medical research institutions, and pharmaceutical and biotechnology companies; as well as independent companies (both for profit and non-profit) that provide biobanking services and sample access to the research community. Increasingly, patients are allowed access to their data (90). There are three main types of human biobanks that exist and are often designed according to the intended research goal. These include population biobanks for the purpose of obtaining biomarkers of population identity and susceptibility, which contain DNA collected from a large cohort of representative healthy donors of a country/region/ethnic group; epidemiological disease-oriented biobanks for research focused on biomarkers of exposure, typically comprising a large collection of biospecimens derived from a healthy exposed cohort/case-control design for the purpose of studying germline DNA or serum markers and large quantities of collected data; and disease-oriented general biobanks (e.g. tumour banks) for research focused on biomarkers of disease, which consist of human biospecimens and their derivatives (e.g. DNA), as well as accompanying clinical data (90).

### Properties of the Biobank

A biobank stores human biospecimens, such as tissue, blood, other body fluids, cells and associated derivatives (e.g. DNA, RNA and protein) collected for a specific (sometimes general) research purpose. These samples are typically stored in low temperature (-80°C) freezers and/or ultralow temperature (-150°C) liquid nitrogen vapour phase tanks for long-term storage, as the low temperatures preserve the quality and integrity of the DNA, RNA, proteins and cellular components. Different sample collection methods and processing conditions are important factors to consider for the purpose of preserving the quality of the sample and are dependent on the type of biospecimen being collected.

Human tissues are usually obtained from surgeries or autopsies immediately following histopathological examination by a pathologist. Processing the collected tissue specimen in neutral-buffered formalin is the most widely accepted clinical practice for the preservation of tissue specimens, such as for the preparation of formalin-fixed paraffin embedded (FFPE) tissue. The “next generation” era has revealed several limitations regarding the use of FFPE samples for molecular, genetics and protein-based studies; with fresh or frozen tissue being a more reliable alternative, particularly for downstream investigations involving whole-genome amplification, whole-genome sequencing, and complementary DNA (cDNA) microarray analysis (91, 92).

Blood is also a common biospecimen that is biobanked for research purposes and is collected in tubes containing preservatives and additives. The type of tube or additive used for collection is dependent on the required blood fraction (e.g. plasma, serum, white blood cells and red blood cells) and the intended downstream research application(s). For instance, ethylenediaminetetraacetic acid (EDTA)-coated collection tubes are generally preferred for DNA- and protein-based assays, whereas Heparin tubes are more suitable for metabolomic studies (91, 93, 94). Furthermore, the optimal storage temperature is dependent on the stability of the specific blood-based biomolecule(s) being investigated, however both -20°C and -80°C storage temperatures are generally considered to be optimal for maintaining the integrity and stability of every blood component type (91, 95, 96).

DNA and RNA derivatives, that have been extracted from the parent tissue and/or blood biospecimens, are also commonly stored in a biobank. The success of downstream molecular analyses and quality of generated data is highly dependent on the integrity of the stored DNA and RNA samples. RNA is particularly more prone to degradation than DNA and the associated yield and quality is influenced by the type of sample it is derived from. For instance, FFPE tissue-derived RNA yield and quality is generally poor in comparison to fresh frozen tissue-derived RNA on account of the cross-linking of nucleic acids that is induced by formalin and the lengthy time interval between tissue resection and fixation (91, 97, 98). To ensure that DNA and RNA quality is maintained, they are typically stored at -80°C without repeated freeze-thaw cycles.

Overall, the quality and success of research aimed to advance health care practices is highly reliant on the correct processing and storage conditions of the biobanked human biospecimens. It is therefore of crucial importance that researchers process and store the biospecimens at the conditions that are most optimal for the intended aims of the research investigation and the type of analyte being measured.

### MM-Specific Biobanks

Some of the most notable MM-specific biobanks worldwide, that consist of an extensive collection of annotated MM patient-derived specimens, include the MesobanK, Cambridgeshire, UK; Cancer of Respiratory Tract (CREST) biorepository, National Cancer Research Institute, Genoa, Italy; the National Centre for Asbestos Related Diseases (NCARD), Perth, Australia; and the Asbestos Diseases Research Institute (ADRI) biobank, Sydney, Australia.

The MesobanK UK in particular, offers an extensive collection of centrally located patient-derived biospecimens. The main objectives of the MesobanK UK is to provide a framework for the systematic collection, curation and quality assurance of well-annotated MM biospecimens that will facilitate high quality basic science, translational and clinical research based on mesothelioma (99). Upon its completion the MesobanK is expected to be comprised of 750 patient tissue microarrays, 300 matched blood and pleural fluid samples, and associated annotated clinical data, as well as 26 newly developed cell lines that can be readily accessed by researchers worldwide upon request (99). It is currently the only MM-specific biobank that offers such a service.

The CREST biorepository was established to investigate the molecular mechanisms and to develop tools and strategies for the primary and secondary prevention of respiratory-tract cancers, which includes both MM and lung cancer. The main goal of the CREST biorepository is to provide a comprehensive resource of respiratory cancer-related biospecimens along with annotated details of corresponding epidemiologic and clinical data in order to facilitate high quality molecular epidemiological and translational studies of respiratory tract cancers, but with particular emphasis on MM (100). The CREST biorepository is particularly beneficial to epidemiological studies focusing on exposure to airborne carcinogens, the identification of subgroups of affected individuals and to estimate cancer risk associated with early molecular events (100). Dating from January 2011, the CREST biorepository was reported to have obtained biospecimens from a total of 1,857 subjects; comprised of 454 lung cancer, 245 MM, 130 other cancer types, and 1,028 healthy controls (101). The biobanked samples sourced from these subjects include tissue biopsies, pleural fluid, saliva, whole blood, plasma, serum and lymphocytes (101).

The NCARD biobank was established to facilitate research focused on the development and implementation of improved clinical outcomes relating to the diagnosis and treatment of asbestos-related diseases, including mesothelioma, for people of Western Australia and worldwide. Since its establishment in 1994, the biobank has obtained samples from 3000 Western Australian subjects, which has enabled the generation of approximately 10,000 biospecimens, such as tissue, blood, pleural fluid and urine; as well as 80 cancer cell lines. External research investigators can obtain biospecimens from the NCARD biobank for use in approved research projects upon approval of a formal application, which is reviewed by the biobank management committee.

The ADRI biobank comprises an extensive collection of MM biospecimens sourced from patients of the Sydney and Greater Western Sydney region. Specifically, these biospecimens are obtained from six different hospital sites, which includes Strathfield Private, Royal Prince Alfred, Concord Repatriation General, Westmead and Sydney Adventist hospitals. The main objective of the ADRI biobank is to provide researchers with high quality biospecimens and annotated data to facilitate research aiming to improve the diagnosis and treatment of MM, and to develop effective preventative measures. The ADRI biobank contains over 2,000 MM patient-derived biospecimens, which includes fresh frozen tissue, FFPE tissue, pleural fluid, blood, primary cells and cell lines; as well as over 12,000 derivatives, which includes tumour DNA, tumour RNA, plasma, buffy coat, serum and red blood cells. The biobank is intended primarily as an in-house resource to be used by ADRI research staff, however external requests for access to samples may be granted for Ethics approved projects in some cases.

Collectively, these biobanks constitute a valuable source of high quality biospecimens and associated clinical data that are critically important for researchers undertaking MM-related investigations. Collaboration across MM biobanks at both national and international levels should be encouraged to promote the sharing of biospecimens and clinical data. Although MM is globally increasing it is still rare in comparison to other cancers and carcinomas which poses a challenge to the collection of biospecimens. Research directed at genetic differences in relation to the causality, progression and response to treatment, has not been adequately addressed so far but can hugely benefit from a collaborative scheme. A more global and interactive MM-specific biobanking network would be particularly beneficial to researchers investigating epidemiological-related factors influencing the disease mechanisms, diagnosis and treatment of MM. From a global perspective, we advocate the establishment of MM biobanks in the many developing countries that continue to use asbestos and which have recently started to diagnose mesothelioma. To this end, we have been engaged in providing international training workshops to improve the recognition and diagnosis of MM (102).

## Expert Commentary and Conclusions

Mesothelioma continues to represent a significant burden on public health worldwide and its incidence is unlikely to decrease in the coming years given the long latency associated with its pathogenesis in asbestos-exposed individuals, combined with continued human exposure to asbestos fibres in the environment. Despite the previous substantial preclinical research efforts that have been devoted to improving our understanding of MM biology with respect to the development of improved diagnostic and therapeutic strategies, clinical practice involving the diagnosis and treatment of MM has remained relatively unchanged over the past few decades and consequently patient prognosis has not improved significantly. Hence, continued basic science research using preclinical models of MM is greatly needed in order to further expand our knowledge of MM biology and to investigate improved diagnostic and treatment strategies. With further investigation of the developmental biology of MM using *in vitro* and *in vivo* models, it will become possible to identify and characterise additional MM-specific molecular targets that can potentially be pursued for the testing and development of improved biomarkers and therapeutic strategies.

As we have summarised, there are a variety of useful preclinical models available to researchers studying MM. Different models, whether they be cell-based or animal-based, have their own intrinsic advantages and disadvantages; no model is perfect. The accuracy and reliability of the generated experimental data is highly dependent on the type of model selected and its suitability to the specific aims or criteria being addressed in the study. Ultimately, MM-based studies that employ accurate preclinical models will stand a better chance at progressing through to clinical trials; particularly studies that are able to reproduce the experimental data using multiple model types. For example, studies that are investigating the efficacy of novel immunotherapeutic agents for MM would only produce clinically relevant and reliable data by utilising a syngeneic subcutaneous and/or orthotopic model, given that they both possess an intact immune system.

Laboratory technology/techniques are constantly evolving, with significant technological advancements having been attained in regards to 3D cell culture, NGS technology and qPCR. These techniques/technology are fundamental to research aiming to explore MM tumour cell response to novel drug treatments, and the identification of novel biomarker candidates that possess valuable diagnostic and/or prognostic qualities. It is crucially important that researchers utilise the most current techniques and technology where possible. For instance, a study examining chemotherapy drug cytotoxicity in a 3D MM cell culture system will likely generate preclinical data that more accurately mimics chemotherapy drug behaviour in the clinical setting compared to the same experiment conducted in a 2D cell culture system. The ddPCR technique was given particular emphasis in this review given that it is a highly reliable and precise PCR technique that has shown emerging potential for the detection and validation of MM-specific biomarkers in recent years. Given the superior sensitivity of this modern PCR technique, it would be highly beneficial to prospective studies aiming to detect and validate novel circulating MM-specific biomarkers that would not normally be detected by other conventional qPCR platforms. Less invasive blood-based biomarkers are particularly lacking for MM and invasive biopsy procedures are still required to attain a definitive diagnosis (103, 104). Hence, prospective research studies aiming to validate and develop a blood-based biomarker panel for MM, through the application of the ddPCR technique, would constitute a significant advancement in the field of MM clinical diagnostics.

Given that mesothelioma is a relatively rare cancer in comparison to other disease types, access to patient biospecimens is somewhat limited and therefore collaborations between expert mesothelioma research centres worldwide should be strongly encouraged to overcome this limitation. Such multi-centre collaborations would enable the sharing of biobanked research specimens and associated data, which would facilitate the development of projects using a large and diverse sample cohort. In turn, these studies would be likely to produce statistically powered data to support the efficacy/validity of novel biomarkers and treatment strategies that would have strong potential to progress through to clinical trials. Furthermore, such multi-centre collaborative studies would enable researchers to more easily afford the high costs typically associated with modern laboratory technologies, such as NGS.

## Author Contributions

YC and BJ conceived the paper, where BJ and YC wrote the paper and all three authors edited the final draft. All authors contributed to the article and approved the submitted version.

## Funding

This review is supported by Regional Collaborations Programme, Australian Academy of Science.

## Conflict of Interest

The authors declare that the research was conducted in the absence of any commercial or financial relationships that could be construed as a potential conflict of interest.

## Publisher’s Note

All claims expressed in this article are solely those of the authors and do not necessarily represent those of their affiliated organizations, or those of the publisher, the editors and the reviewers. Any product that may be evaluated in this article, or claim that may be made by its manufacturer, is not guaranteed or endorsed by the publisher.
